# Cream Cheese-Derived *Lactococcus chungangensis* CAU 28 Modulates the Gut Microbiota and Alleviates Atopic Dermatitis in BALB/c Mice

**DOI:** 10.1038/s41598-018-36864-5

**Published:** 2019-01-24

**Authors:** Jong-Hwa Kim, Kiyoung Kim, Wonyong Kim

**Affiliations:** Department of Microbiology, Chung-Ang University College of Medicine, Seoul, Korea

**Keywords:** Microbiome, Nutritional supplements

## Abstract

Atopic dermatitis (AD) has a drastic impact on human health owing to complex skin, gut microbiota, and immune responses. Some lactic acid bacteria (LAB) are effective in ameliorating AD; however, the alleviative effects of dairy products derived from these LAB remain unclear. In this study, the efficacies of *Lactococcus chungangensis* CAU 28 (CAU 28) cream cheese and *L*. *chungangensis* CAU 28 dry cells were evaluated for treating AD in an AD mouse model. Overall, CAU 28 cream cheese administration was more effective against AD than *L*. *chungangensis* CAU 28 dry cells. Faeces from CAU 28 cream cheese-administered mice had increased short chain fatty acid, butyrate, acetate, and lactic acid levels, as well as butyrate-producing bacteria, including *Akkermansia*, *Bacteroides*, *Lactobacillus*, and *Ruminococcus*. Furthermore, oral CAU 28 cream cheese administration resulted in regulatory T cell (Treg)-mediated suppression of T helper type 2 (Th2) immune responses in serum and mRNA expression levels in the ileum. Oral CAU 28 cream cheese further reduced IgE levels, in addition to eosinophil and mast cell numbers. Therefore, CAU 28 cream cheese administration induced a coordinated immune response involving short-chain fatty acids and gut microbiota, indicating its potential for use as a supplement for AD mitigation.

## Introduction

Recently, interest in fermented foods, particularly cheese, kefir, yogurt, and kimchi has risen because of their potential for health-promotion in a manner indirectly attributable to the food materials themselves^1^. Intake of fermented foods has the potential to increase gut microbes and daily consumption of fermented food products can temporarily introduce transient microbes into the indigenous gut microbiota^2,3^. Interestingly, the consumption of fermented dairy products incorporating beneficial bacteria modifies the gut microbiota toward an increase in production of butyrate (a short-chain fatty acid [SCFA] metabolite produced by gut microbiota) relative to that induced by chemically acidified milk^4^.

Cheese is one of the most variable fermented milk-based food products and comprises biochemically and biologically dynamic matrices influenced by microbial activity and composition^5^. Moreover, cheese is useful as a delivery system to introduce probiotics into the gastrointestinal tract and can reduce the likelihood of a highly acidic gut environment, thereby promoting probiotic survival^6^. Among cheeses, cream cheese is a soft fresh cheese with a slightly buttery flavour, prepared using pasteurized milk and lactic acid bacteria (LAB) as the starter culture^7^.

LAB are a group of gram-positive organisms comprising species of *Lactococcus*, *Lactobacillus*, *Leuconostoc*, *Pediococcus*, and *Streptococcus*^8^. Some LAB strains, such as *Lactobacillus* spp., *Lactococcus lactis*, and *Bifidobacterium* spp. are important in maintaining gut homeostasis and provide health benefits through balancing T helper type 1 (Th1) and T helper type 2 (Th2) immune responses^9,10^. Among LAB, *Lactococcus* spp. are used as starter cultures for the production of cheese and fermented milk-based products; however, their probiotic function has frequently been underestimated because of an assumption that they cannot survive in the gastrointestinal tract^11^.

The specific strain, *Lactococcus chungangensis* CAU 28, isolated from non-dairy environments is the sixth member of genus *Lactococcus*^12^. This strain was previously examined by transcriptomic analysis for the presence of functional genes, including those encoding cystathionine β-lyase (*MetC*), O-acetylserine sulfhydrylase (*CysK*), alcohol dehydrogenase (*ADH*), aldehyde dehydrogenase (*ALDH*), and activities of other enzymes, such as amylase, proteinase, and lipase^13–16^. *L*. *chungangensis* CAU 28 has been shown to be beneficial for the treatment of AD^17^; however, that study focused solely on the application of *L*. *chungangensis* CAU 28 in Nc/Nga mice and did not reveal any underlying mechanism of action.

Atopic dermatitis (AD) is a chronic inflammatory skin disease caused by a variety of genetic, environmental, and immunological factors, alongside exposure to microorganisms or allergens^18^. Abnormal Th2-type immune responses associated with skin damage or exposure to microbial stimuli are proposed as major mechanisms underlying AD^19^. Traditionally, topical corticosteroids, tacrolimus, and antihistamines have been used as basic treatments for AD to reduce inflammation; however, these approaches can trigger side effects and simply alleviate the symptoms rather than the underlying aetiology^20,21^. The applications of gut microbiota in disease treatment has garnered an increasing level of interest in recent years^22^. Numerous studies have reported potential beneficial effects of probiotics, including the specific strain, *Lactobacillus rhamnosus* GG. However, the applications of isolated bacterial strains with probiotic effects are limited, while the use of probiotic mixtures may have negative effects or may yield data that is difficult to interpret^23–25^.

It is important to identify treatments that target AD without triggering systemic side effects. To address this, recent studies on probiotics and gut microbiota, which have important nutrient and immune functions, have focused on the prevention or treatment of AD^26^. In the present study, a comprehensive investigation was conducted to explore the potential alleviative effects of cream cheese-derived *L*. *chungangensis* CAU 28 with *L*. *chungangensis* CAU 28 dry cells and bepotastine besilate. Furthermore, the effects of these supplements were evaluated based on analyses of immune response modulation and alterations in the gut microbiota in an AD mouse model.

## Results

### Effects of *L*. *chungangensis* CAU 28 cream cheese on mouse gut microbiota profiles

Five-week-old female BALB/c mice (n = 50) were randomly assigned to five groups (n = 10/group) as follows: (1) negative control group, which included mice that were not subjected to ovalbumin (OVA) sensitization and orally administered with phosphate-buffered saline (PBS); (2) positive control group, sensitized with OVA and orally administered with PBS; (3) bepotastine besilate (BB) group, sensitized with OVA and orally administered with BB (an antihistamine); (4) *L*. *chungangensis* CAU 28 (CAU 28) group, sensitized with OVA and orally administered with freeze-dried *L*. *chungangensis* CAU 28; (5) *L*. *chungangensis* CAU 28 (CAU 28) cream cheese group, sensitized with OVA and orally administered with cream cheese prepared using *L*. *chungangensis* CAU 28.

**Figure 1:**
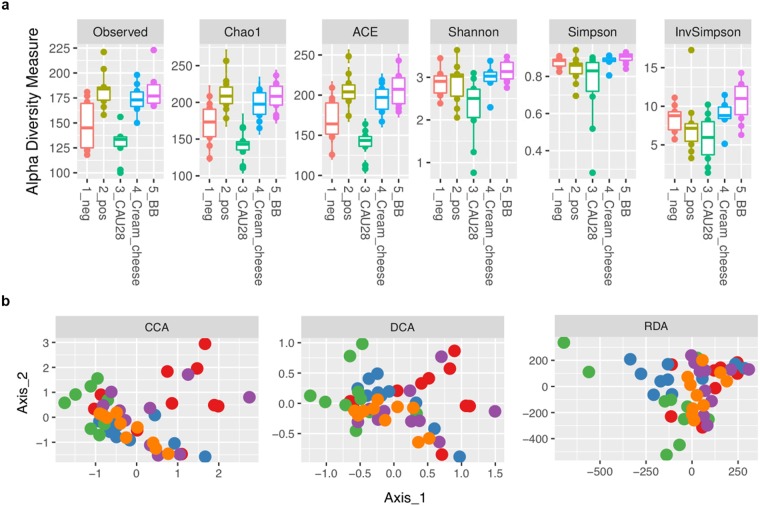
Alpha diversity and similarity in microbial communities. (**a**) Species richness estimates: observed number of species, Chao1 and ACE indices. Diversity estimates: Shannon, Simpson, and InvSimpson indices. (**b**) Distances between groups were determined via constrained correspondence analysis, detrended correspondence analysis, and redundancy analysis, based on similarity of operational taxonomic units (OTUs) (>97% OTU similarity). All indices were statistically significant (*p* < 0.05). neg, negative control group; pos, positive control group; cream, CAU 28 cream cheese; BB, bepotastine besilate. Red, negative control group; blue, positive control group; green, CAU 28; purple, CAU 28 cream cheese; orange, bepotastine besilate.

To identify changes in microbial diversity, bacterial DNA was isolated from 16-week-old faecal samples and 16 S rDNA was PCR-amplified and subjected to Illumina-based high-throughput sequencing. In total, 5,035,756 bacterial sequence reads were generated, with an average of 100,715 sequence reads and an average read length of 598 bp (±8.8 bp) per sample. The diversity, evenness, and richness of the bacterial community was statistically determined from sequencing data based on the observed number of species, Chao1, ACE, Shannon, Simpson, and InvSimpson indices. The observed number of species, Chao1, and ACE indices indicated greater species richness in the CAU 28 cream cheese group than in the other groups (*p* < 0.05; Fig. 1a). Moreover, the CAU 28-derived cream cheese group exhibited greater diversity and evenness in gut microbiota compared with the positive control group, as indicated by Shannon, Simpson, and InvSimpson indices (*p* < 0.05; Fig. 1a; Table S1).

Furthermore, to assess the differences in microbiota profiles, constrained correspondence analysis (CCA), detrended correspondence analysis (DCA), and redundancy analysis (RDA) were performed. The results of CCA and DCA indicated that the relationship between the difference of microbial community, and the CAU 28 cream cheese group showed significant correlations with negative group (*p* < 0.05). According to the results of these correlation analysis, RDA result also showed that negative control, CAU 28 cream cheese, and BB were clustered into significant distinct from others (*p* < 0.05).

Overall, the results demonstrated a significant difference between the CAU 28 cream cheese group and the positive control group (*p* < 0.05; Fig. 1b). Together, the present results indicate that the gut microbiota of the CAU 28 cream cheese group was significantly different from that of the positive control group.

**Figure 2:**
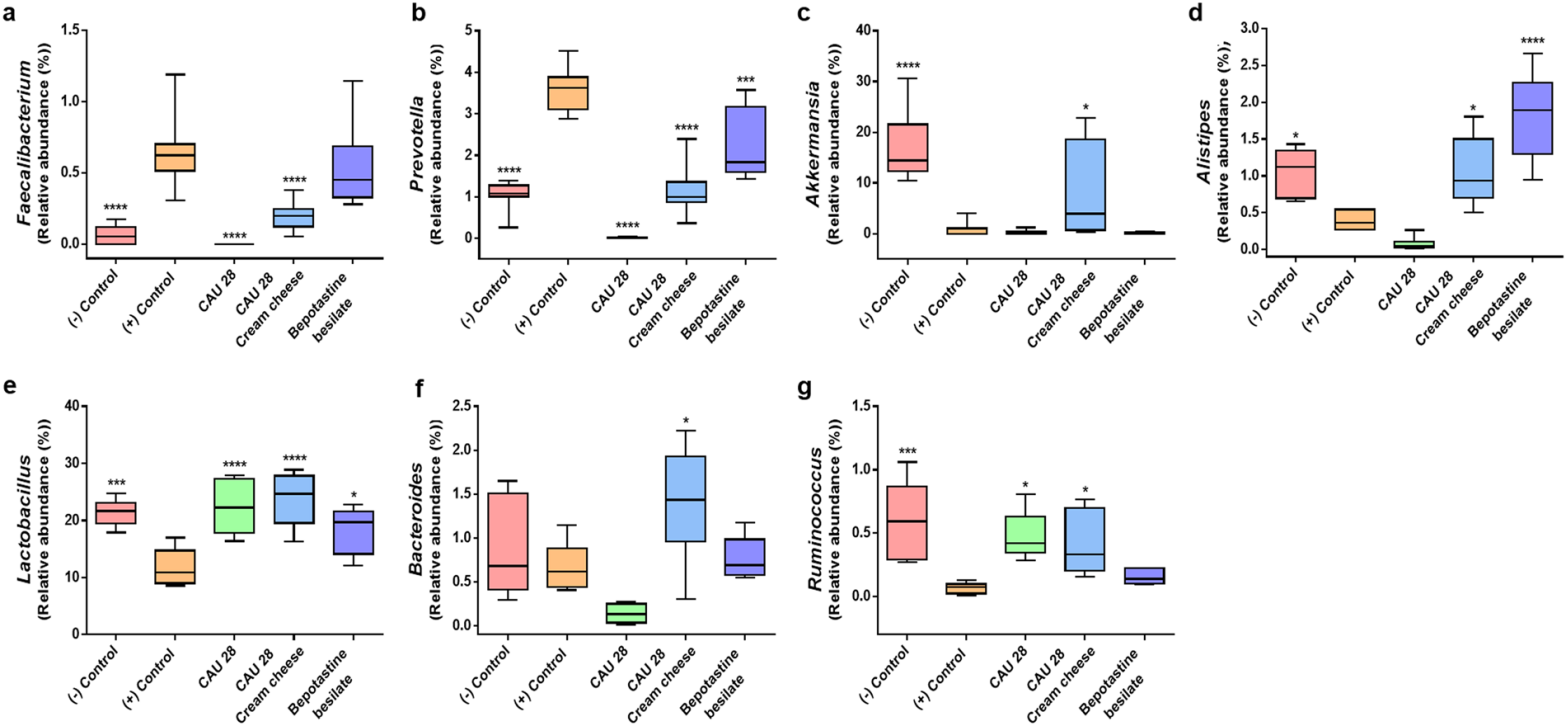
Gut microbial communities in the atopic dermatitis (AD) mouse model. Relative abundance of genera (**a–g**). The box plots show the frequencies of statistically significant genera. Significance marks (*p < 0.05; ***p < 0.0005; ****p < 0.0001) indicate differences relative to the means for the positive control group, determined using one-way ANOVA.

Fourteen bacterial phyla were identified in the mice, of which the three most predominant were Firmicutes, Bacteroidetes, and Verrucomicrobia (Fig. S1). Sixty bacterial families were identified in the experimental groups, among which *Veillonellaceae*, *Prevotellaceae*, *Verrucomicrobiales*, *Rikenellaceae*, *Bacteroidales*, *Bacteroidaceae*, and *Lactobacillaceae* were most prominent (Fig. S2). These phyla and families included 129 genera, seven of which exhibited significant differences in abundance among the experimental groups (Fig. 2 and Table S2). Within phylum Firmicutes, the predominance of family *Faecalibacterium* was significantly lower in the negative control group, CAU 28, and CAU 28 cream cheese groups than in the positive control group (*p* < 0.0001). Conversely, *Ruminococcus* displayed a significantly greater predominance in the negative control (*p* < 0.01), CAU 28 (*p* < 0.05), and CAU 28 cream cheese groups (*p* < 0.05) than in the positive control group. Moreover, in this phylum, family *Lactobacillus* was more abundant in the negative control group (*p* < 0.001), CAU 28 (*p* < 0.0001), CAU 28 cream cheese group (*p* < 0.0001), and BB group (*p* < 0.05) than in the positive control group. In phylum Bacteroidetes, the predominance of family *Prevotella* was lower in the negative control (*p* < 0.0001) and treatment groups (*p* < 0.0001) than in the positive control group. In contrast, *Alistipes* was more abundant in the negative control (*p* < 0.05), CAU 28 cream cheese (*p* < 0.05), and BB groups (*p* < 0.0001) than in the positive control group. Moreover, *Bacteroides* were present at significantly higher levels in the CAU 28 cream cheese-treated group (*p* < 0.05) than in the positive control group. *Akkermansia* (phylum Verrucomicrobia) was more abundant in the negative control (*p* < 0.0001) and CAU 28 cream cheese groups (*p* < 0.05) than in the positive control group. These present results indicate that the gut microbiota and the abundance of their genera differed between the CAU 28 cream cheese and positive control groups.

### Treatment of AD mice with *L. chungangensis* CAU 28 cream cheese led to upregulation of SCFA levels

**Figure 3:**
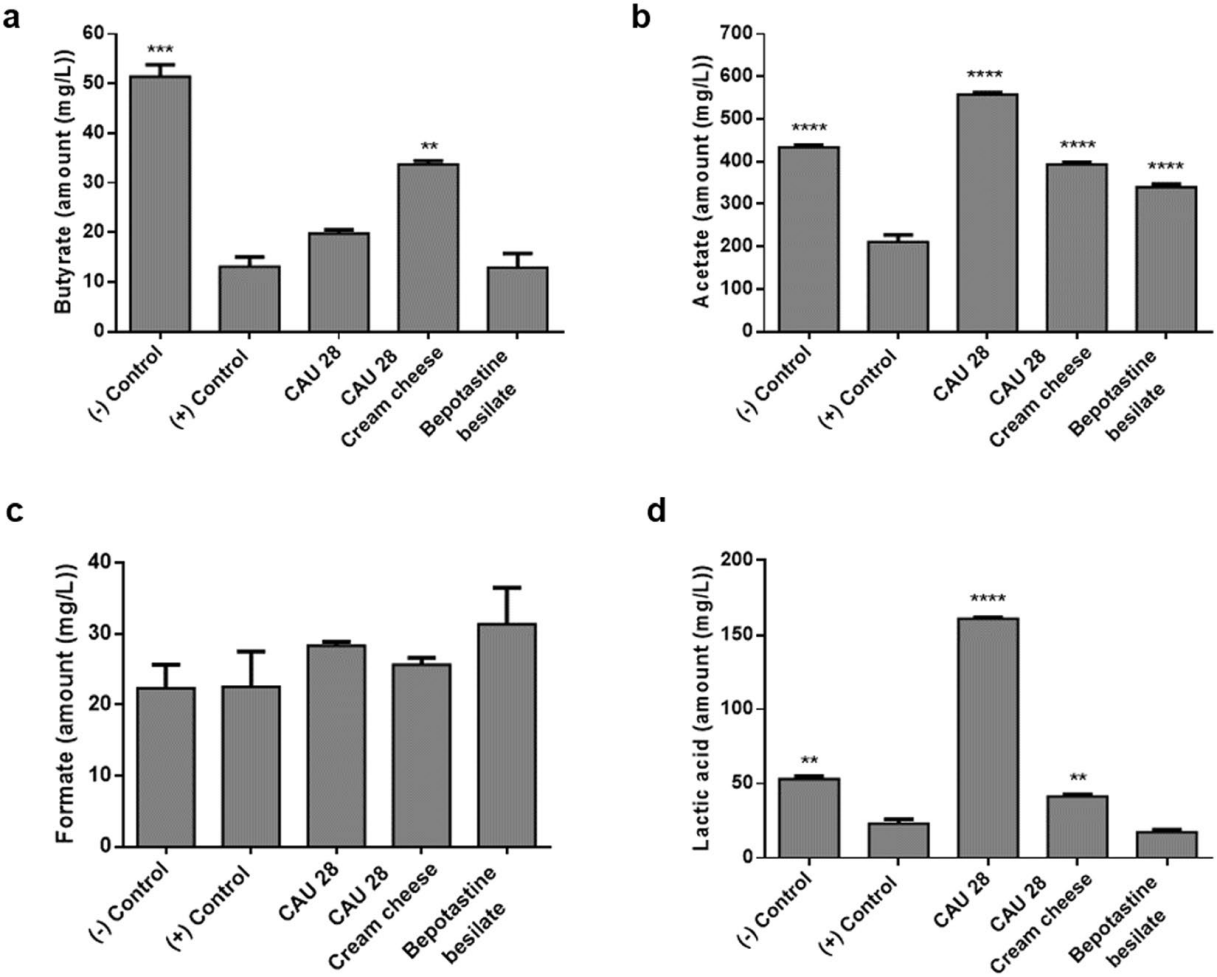
Effect of oral administration of *Lactobacillus chungangensis* CAU 28 cream cheese on short-chain fatty acids (SCFAs). Faecal SCFAs including (**a**) butyrate, (**b**) acetate, (**c**) formate, and, (**d**) lactic acid were analysed via high-performance liquid chromatography. Significance marks (**p < 0.005; ***p < 0.0005; ****p < 0.0001) indicate differences relative to the means for the positive control group determined using one-way ANOVA.

To compare gut microbiota metabolite production in the experimental groups, faecal samples were examined for acetate, butyrate, and formate using high-performance liquid chromatography (HPLC; Fig. 3). SCFA, butyrate and acetate, and lactic acid levels were lower in the positive than in the negative control group. Faecal samples from the CAU 28 and CAU 28 cream cheese groups contained significantly higher levels of acetate and lactic acid, and those from the CAU 28 cream cheese group had significantly higher levels of butyrate than the positive control group.

### Effect of *L*. *chungangensis* CAU 28 cream cheese on cytokine levels and mRNA expression levels

**Figure 4:**
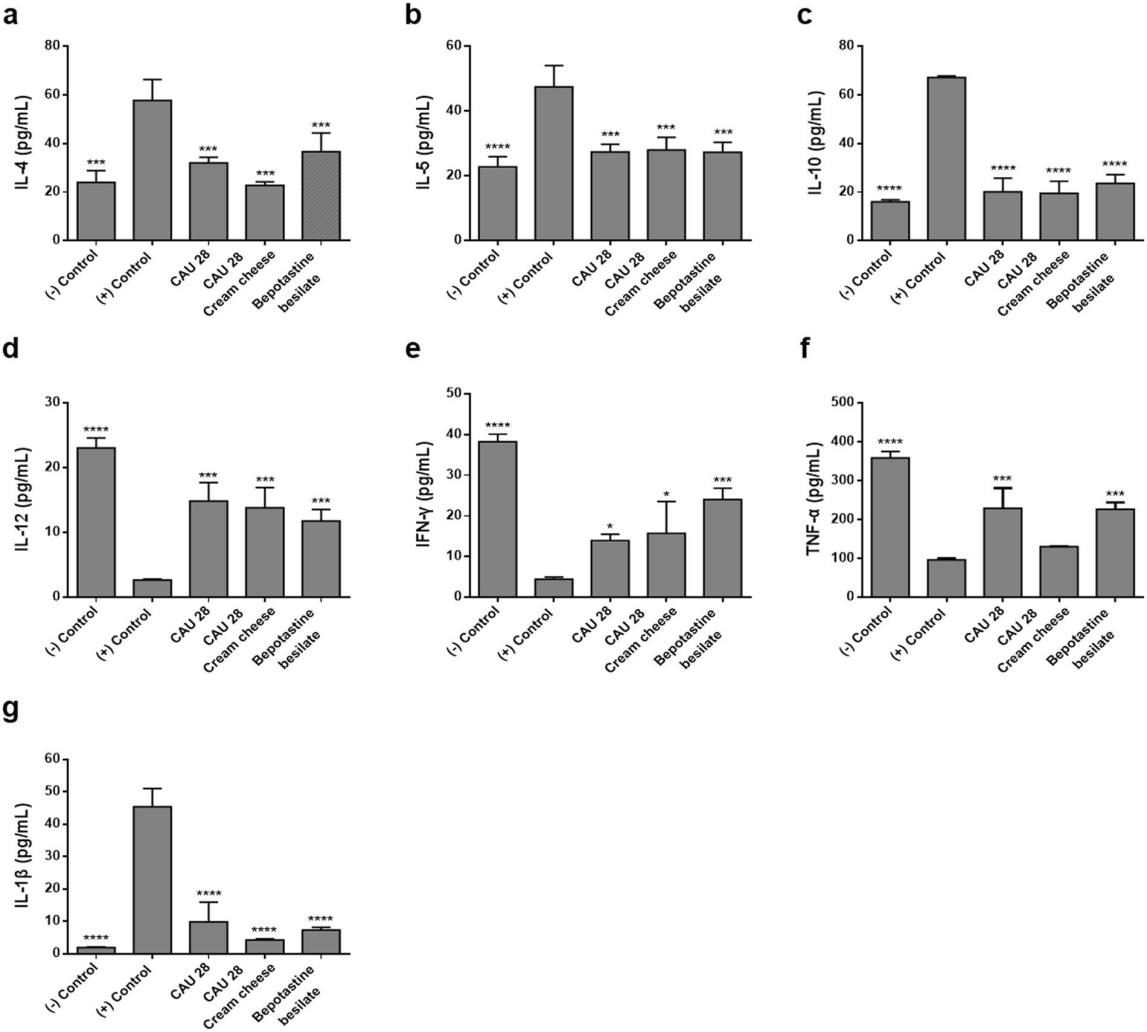
Effect of oral administration of *Lactobacillus chungangensis* CAU 28 cream cheese on cytokine levels. Levels of cytokines including (**a**) IL-4, (**b**) IL-5, (**c**) IL-10, (**d**) IL-12, (**e**) IFN-γ, (**f**) TNF-α, and (**g**) IL-1β were determined via an enzyme-linked immunosorbent assay. Significance marks (*p < 0.05; ***p < 0.0005; ****p < 0.0001) indicate differences relative to the means of the positive control group determined using one-way ANOVA.

**Figure 5:**
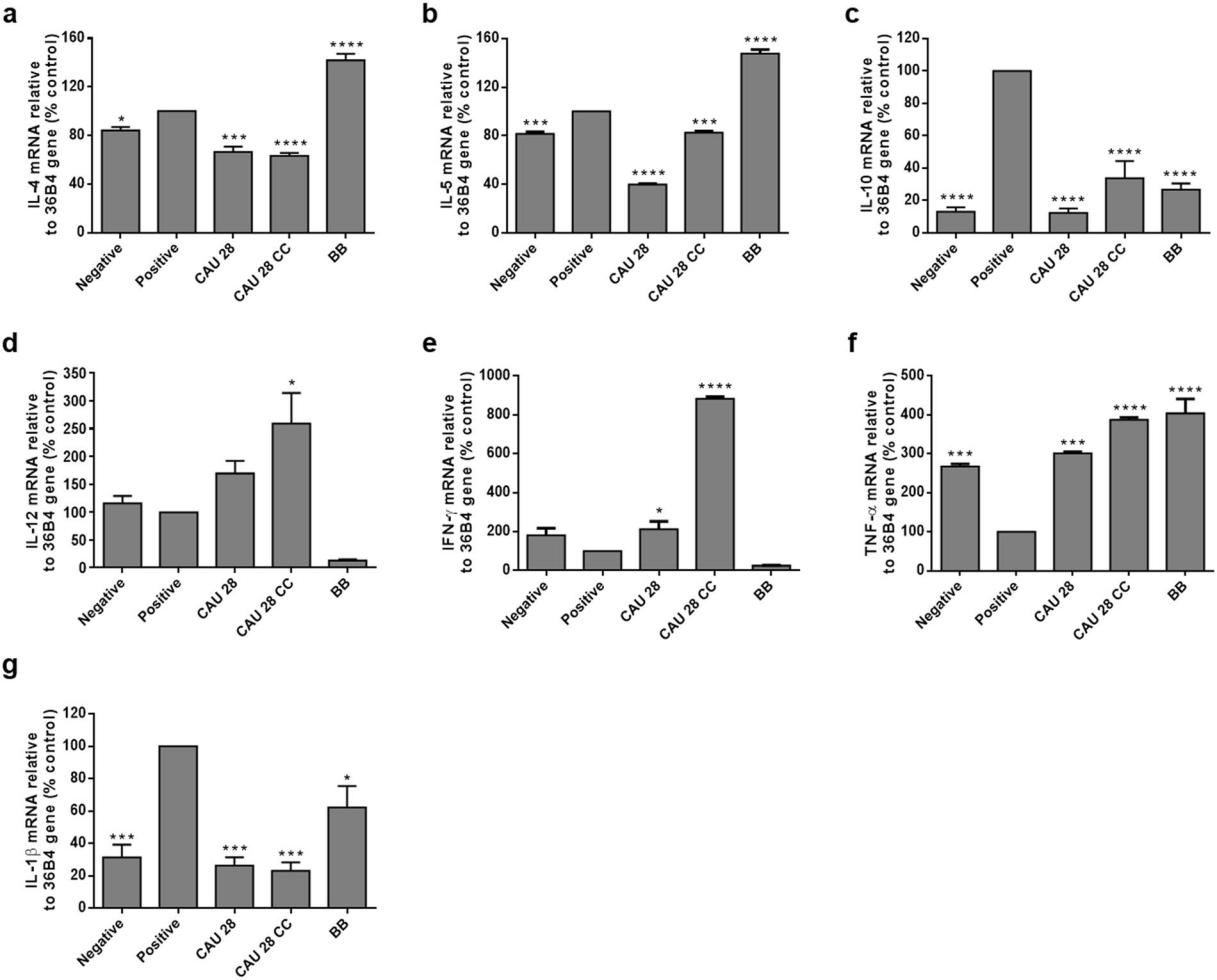
Effect of oral *Lactobacillus chungangensis* CAU 28 cream cheese administration on mRNA expression. Target mRNA expression levels including those of (**a**) IL-4, (**b**) IL-5, (**c**) IL-10, (d) IL-12, (e) IFN-γ, (f) TNF-α, and (g) IL-1β were determined via real-time polymerase chain reaction. Significance marks (*p < 0.05; ***p < 0.0005; ****p < 0.0001) indicate differences relative to the means of the positive control group determined using one-way ANOVA.

To examine the effects of CAU 28 and CAU 28 cream cheese on cytokine production, serum cytokine concentrations were examined (Fig. 4). The serum concentration of cytokines produced by regulatory T cells (Tregs), such as IL-10 and IL-1β, increased in the positive control group compared with the CAU 28, CAU 28 cream cheese, and BB groups. Serum concentrations of IL-4 and IL-5, which are important Th2 cytokines, were significantly lower in the CAU 28, and CAU 28 cream cheese groups than in the positive control group; however, levels of the Th1 cytokines, IL-12, IFN-γ, and TNF-α, were lower in the positive control group than in the CAU 28 and CAU 28 cream cheese groups. Furthermore, to evaluate the effect of CAU 28 and CAU 28 cream cheese on the Th1/Th2 balance in ileum, Th1, Th2, and Treg type response-related inflammatory mRNA expression was examined (Fig. 5). Most mRNA expression levels of Th2 cytokines (IL-4 and IL-5) and Treg cytokines (IL-10 and IL-1β) were significantly downregulated in the CAU 28, and CAU 28 cream cheese groups than in the positive control group, and the levels of the Th1 cytokines (IL-12, TNF-α and IFN-γ) increased following stimulation with CAU 28 cream cheese. These results indicate that cream cheese prepared using *L*. *chungangensis* CAU 28 enhances the Treg-mediated suppression of Th2 immune responses.

### Evaluation of the correlation between bacteria types and the expression of immune co-stimulatory molecules by flow cytometric analysis

**Figure 6:**
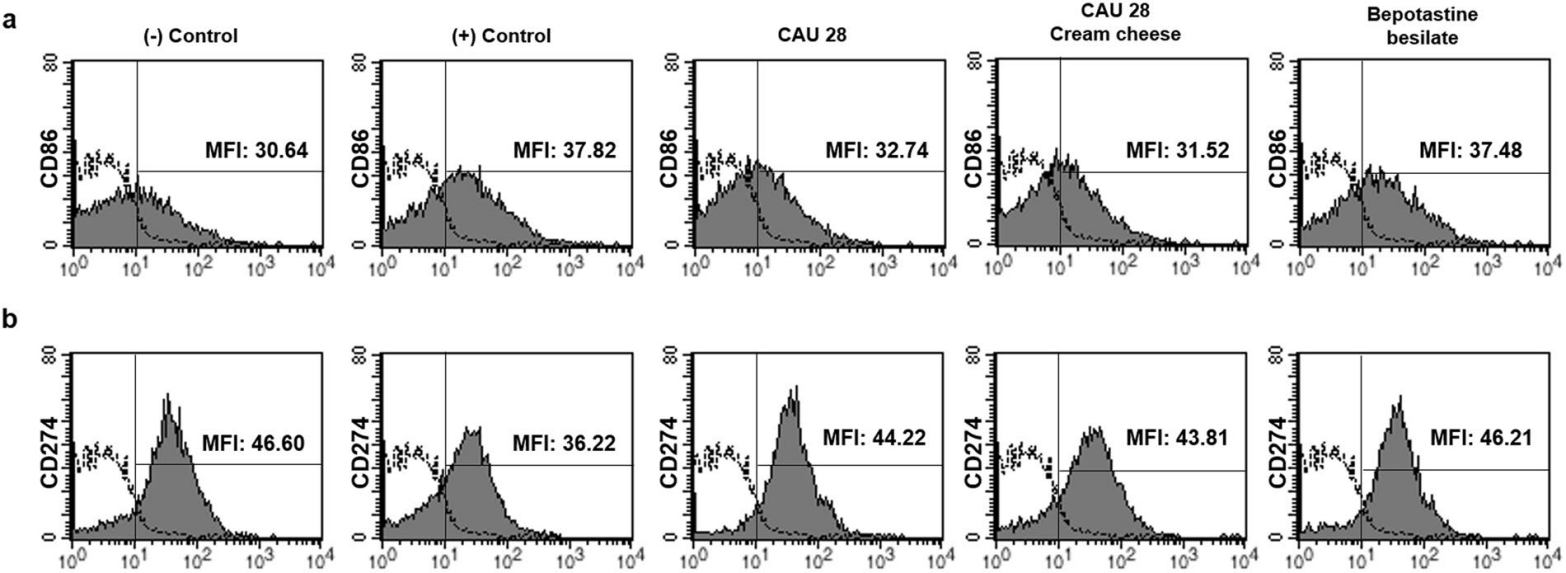
The effect of oral *Lactobacillus chungangensis* CAU 28 cream cheese administration on T cell activation. T cell activation was evaluated via flow cytometric analysis. Filled histograms indicate the reduction in the mean florescence intensity (MFI) value of cells expressing (**a**) CD86 and an increase in the MFI value of cells expressing (**b**) CD274, relative to positive control mouse splenocytes. Unfilled histogram, isotype antibody controls.

To evaluate and compare the expression levels of the immune co-stimulatory molecules, CD80, CD86, CD273, and CD274, splenocytes were isolated from the treatment groups for flow cytometric analysis. Compared with the control group, CD 86 expression (MFI; mean florescence intensity) was significantly lower in the CAU 28 cream cheese-treated group (31.52% ± 1.33%, *p* < 0.05) (Fig. 6). Additionally, compared with the positive control group, CD 274 expression was significantly higher in the CAU 28 (44.22% ± 1.52%, *p* < 0.005), CAU 28 cream cheese (43.81% ± 3.23%, *p* < 0.05), and BB (46.21% ± 4.20%, *p* < 0.001) groups (Fig. 5). No significant changes in CD80 and CD273 expression levels were observed in any of the groups.

**Figure 7:**
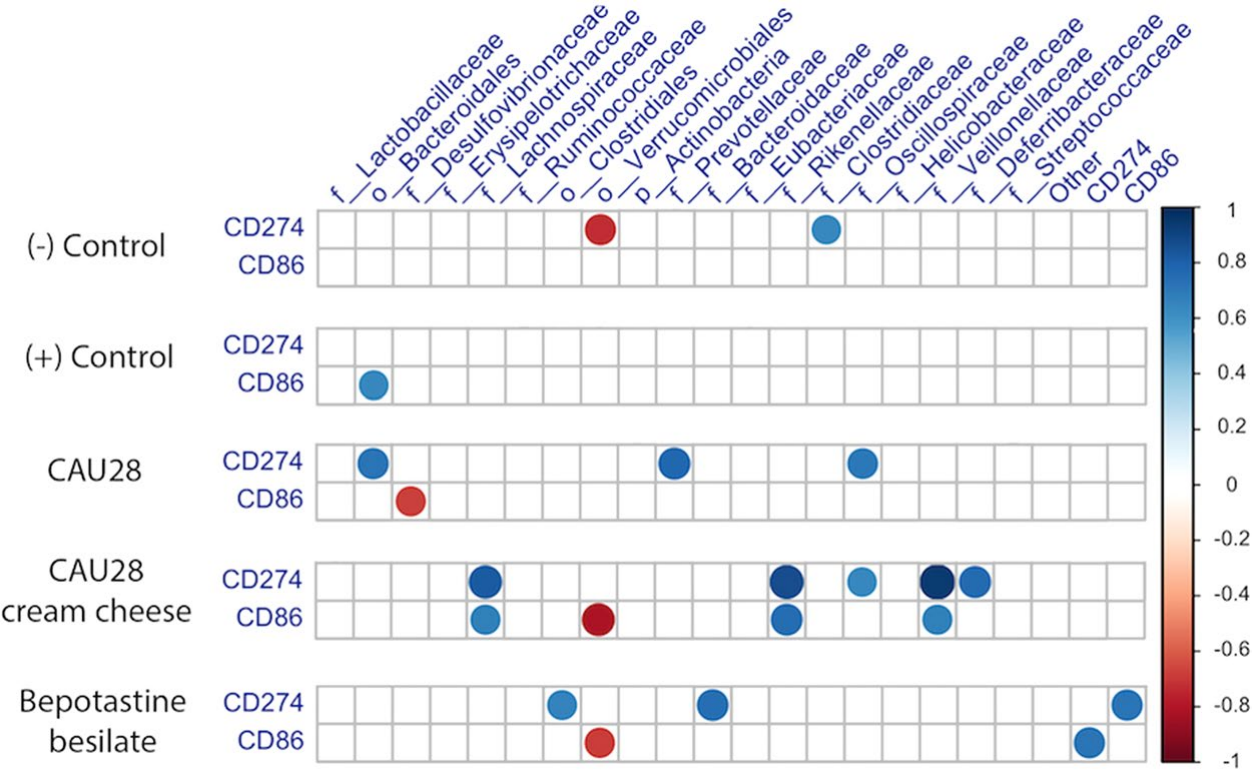
Correlations between T cell activation and bacterial taxonomy at the family level. Spearman’s non-parametric rank correlation matrices for representative bacterial families and markers of T cell activation (CD86 and CD274). All correlations were significant (*p* < 0.05). The colour intensity represents the strength of negative (red circles, closer to −1) or positive (blue circle, closer to 1) correlations between the bacterial taxa and T cell activation.

At the family level, bacterial taxa formed a complex network relative to the expression of co-stimulatory molecules CD86 and CD274 (Fig. 7). There was a positive correlation between CD 274 and the *Bacteroidales*, *Prevotellaceae*, and *Oscillospiraceae* families (*p* < 0.05) in the CAU 28 group; while there was negative correlation between *Desulfovibrionaceae* and CD 86 in this group (*p* < 0.05). CD86 expression levels were significantly correlated with the presence of several bacterial families, including *Verrucomicrobiales*, while those of CD274 correlated significantly with families *Veillonellaceae*, *Rikenellaceae*, *Oscillospiraceae*, and *Deferribacteraceae* in the CAU 28 cream cheese group (*p* < 0.05).

### Effects of *L*. *chungangensis* CAU 28 cream cheese on blood and serum IgE levels

**Figure 8:**
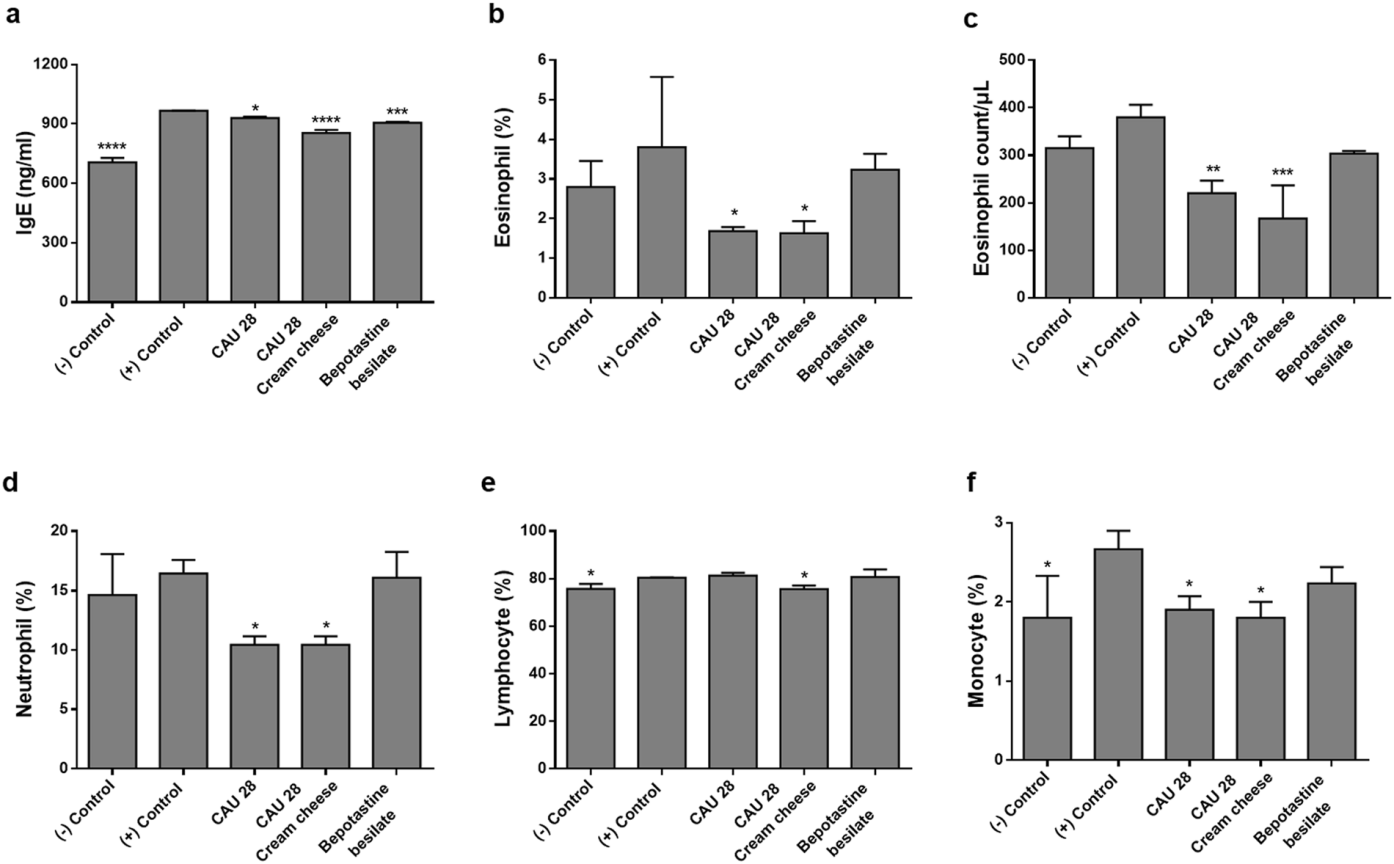
Effect of oral *Lactobacillus chungangensis* CAU 28 cream cheese administration on blood parameters. (**a**) Serum IgE levels were measured via an enzyme-linked immunosorbent assay to determine the occurrence of an allergic response. Inflammation was evaluated via whole blood analysis, including (**b**) eosinophil percentages (**c**) eosinophil count, and (**d**) neutrophil, (**e**) lymphocyte, and (**f**) monocyte percentages. Significance marks (*p < 0.05; **p < 0.005; ***p < 0.0005; ****p < 0.0001) indicate differences between means relative to the positive control group determined using one-way ANOVA.

Serum IgE levels were elevated in the positive control group and significantly decreased in the CAU 28, CAU 28 cream cheese, and BB groups (Fig. 8a). Moreover, eosinophil, neutrophil, lymphocyte, and monocyte percentages in whole blood were greater in the positive control group than in the negative control group, similar to the trend observed for eosinophils (Fig. 8b–f). Eosinophil count, and eosinophil, neutrophil, and monocyte percentages were significantly lower in the CAU 28 and CAU 28 cream cheese groups than in the positive control group. Moreover, lymphocytes were significantly less abundant in the CAU 28 cream cheese group than in the CAU 28 and BB groups.

### Effect of *L*. *chungangensis* CAU 28 cream cheese on skin and gut inflammation

**Figure 9:**
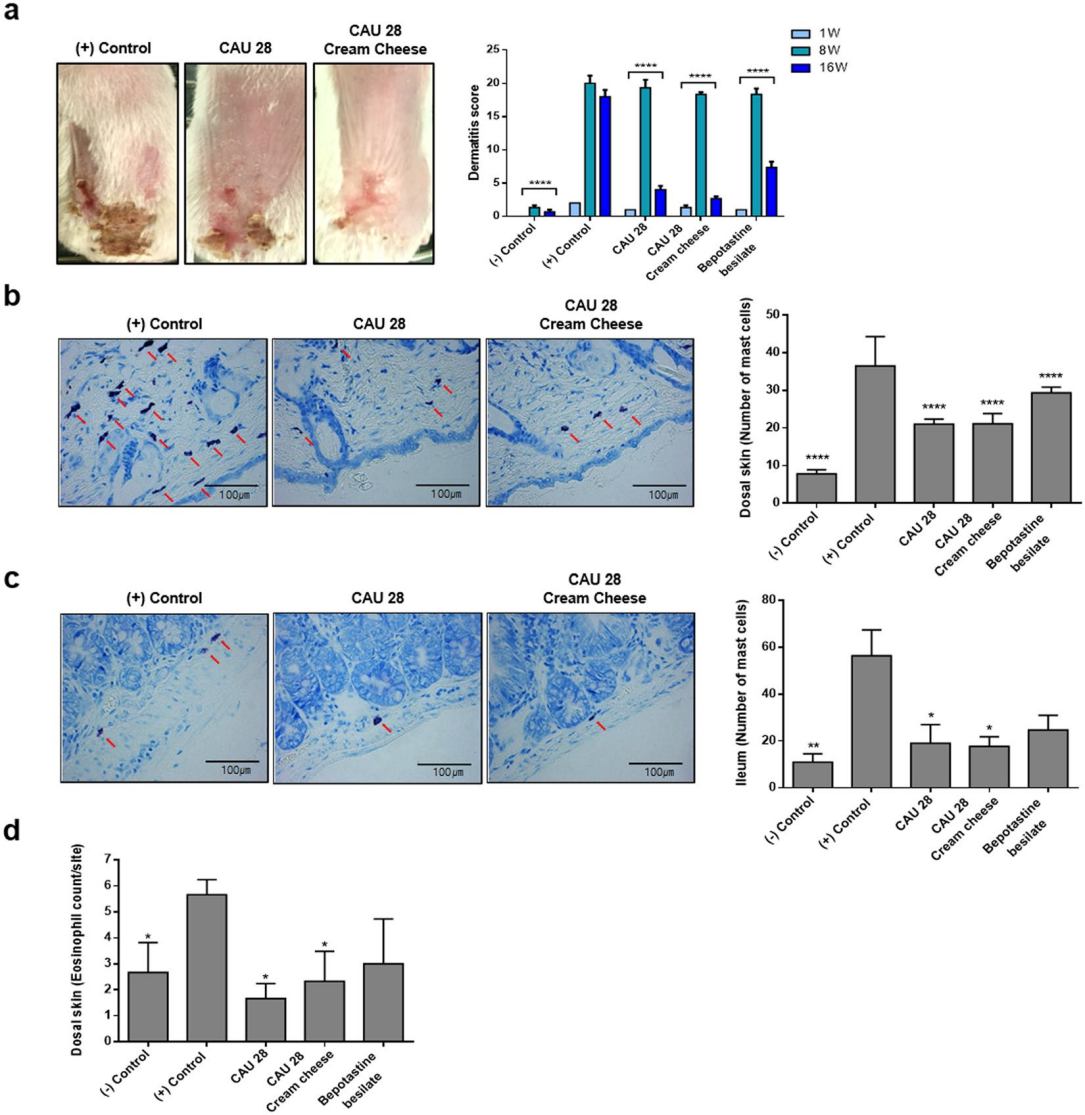
Effect of oral administration of *Lactobacillus chungangensis* CAU 28 cream cheese on atopic dermatitis (AD) skin lesions in mice. (**a**) AD lesions on the dorsal skin were evaluated, and dermatitis scores were calculated from the sum of scores for three symptoms: erythema, dryness, and scratching. Paraffin blocks of (**b**) dorsal skin and (**c**) ileum sections were stained with Toluidine Blue and mast cells were enumerated microscopically. Scale bars = 100 μm. (**d**) Numbers of eosinophils in dorsal skin were quantified microscopically after staining with Congo Red. Significance marks (*p < 0.05; **p < 0.005; ****p < 0.0001) indicate differences relative to the means of the positive control group determined using one-way ANOVA.

To investigate the therapeutic effects of *L*. *chungangensis* CAU 28 cream cheese, mice were sensitized with ovalbumin (OVA) to induce an inflammatory response. OVA-sensitized mice treated with CAU 28, CAU 28 cream cheese, and BB were evaluated via dermatitis scoring and histological analysis. Dermatitis scores were significantly lower in OVA-sensitized mice treated with CAU 28, and CAU 28 cream cheese than in the positive control group (Fig. 9a). This result indicates that CAU 28, and CAU 28 cream cheese had a pronounced effect on the recovery of skin lesions in these mice.

Local mast cell infiltration into dorsal skin and ileal lesions was examined and quantified via Toluidine Blue staining. This analysis revealed that mast cell accumulation at both lesion sites was suppressed in OVA-sensitized mice treated with CAU 28 and CAU 28 cream cheese (Fig. 9b,c). In addition, the number of eosinophils was significantly lower in OVA-sensitized mice treated with CAU 28, CAU 28 cream cheese, and BB than in the positive control group. Congo Red staining revealed reduced eosinophil infiltration in the dorsal skin lesions of OVA-sensitized mice treated with of CAU 28, CAU 28 cream cheese, and BB (Fig. 9d). Together, these results indicate that CAU 28 and CAU 28 cream cheese can suppress mast cell and eosinophil infiltration into dorsal skin and ileal lesions in OVA-sensitized mice.

## Discussion

Numerous studies have investigated the potential therapeutic effects of probiotics in AD. These studies have primarily used beneficial *Lactobacillus* spp. and *Bifidobacterium* spp. strains^27^. The most frequently used strain in these studies was *L*. *rhamnosus* GG, and a number of studies have suggested that this strain is beneficial in preventing the onset AD, either alone or as part of a probiotic mixture^28,29^; however, several other studies reported that the *L*. *rhamnosus* GG strain, either alone or as part of mixed cultures, has limited effects^24,25^ or confers no significant benefit^30^ in the treatment of AD. Recently, it was reported that consumption of yogurt, which contains *Lactobacillus* spp., is inversely associated with AD and affects the gut microbiota; nevertheless the mechanisms underlying these phenomena remain poorly understood^31^. Thus, the utility of probiotics in treating AD remains unclear.

SCFAs have emerged as important biological indicators for both maintenance of health and disease pathogenesis^32^. Gut microbiota are known to synthesize SCFAs^33^, which are potential mediators of the activity and composition of the gut microbiota^34^; hence, these primary metabolic end products of gut microbiota are important for gut homeostasis and health. Furthermore, SCFAs (namely butyrate, acetate, and propionate) exert physiological effects^4,35^. Among SCFAs, butyrate is important in innate immunity, maintenance of gut health, exerts anti-inflammatory effects, and stimulates Treg differentiation^36^. In the present study, high SCFA (butyrate and acetate) and lactic acid levels were associated with changes in the gut microbiota in the CAU 28 and CAU 28 cream cheese groups. These groups contained an abundance of the genera *Lactobacillus* (family *Lactobacillaceae*), *Bacteroides* (family *Bacteroidaceae*) (except for the CAU 28 group), *Ruminococcus* (family *Clostridiaceae*), and *Akkermansia* (family *Verrucomicrobiales*) (except for the CAU 28 group), while members of genus *Faecalibacterium* (family *Clostridiaceae*) were less abundant. A low capacity for SCFA production (butyrate and propionate) is associated with abundance of specific gut bacteria, such as *Faecalibacterium* spp. in AD^37^, and an abundance of members of genera *Lactobacillus*, *Bacteroides*, *Ruminococcus*, and *Akkermansia* is associated with the maintenance of gut health via production of lactate and other SCFAs^38,39^. The results presented here demonstrate an abundance of butyrate-producing bacteria associated with an increase in metabolic activity. Moreover, these data suggest that the beneficial effects of *L*. *chungangensis* CAU 28 cream cheese result from interactions between the gut microbiota and SCFAs in AD.

In the present study, to elucidate the mechanisms underlying their role in AD treatment, the effects of *L*. *chungangensis* CAU 28 cream cheese and freeze-dried *L*. *chungangensis* CAU 28 on the immune response and gut microbiota were investigated in BALB/c mice. CD86 expression was associated with bacterial taxa at the family level (*Verrucomicrobiales*) and CD274 expression was associated with five bacterial families (*Veillonellaceae*, *Rikenellaceae*, *Oscillospiraceae*, and *Deferribacteraceae*) in the CAU 28 cream cheese group. In addition, CD86 and CD274 were significantly down- and upregulated, respectively, in the CAU 28 cream cheese group. CD86 is a co-stimulatory molecule for T-cell activation, which is expressed on antigen-presenting cells and is regulated in response to IL-10^40^, while CD274, which is associated with impaired immune regulatory function, is downregulated in response to IFN-γ^41^. Furthermore, mice treated with CAU 28 and CAU 28 cream cheese displayed significantly lower levels of Th2 (IL-4 and IL-5) and Treg (IL-10) cytokines, and higher levels of Th1 cytokines (IL-12, TNF-α, and IFN-γ) than the positive control group. Mice treated with bepotastine besilate displayed significant suppression of Th2 cytokines in the serum; however, these cytokines were upregulated in the intestine, in comparison with the positive control group. The effects of bepotastine besilate in AD are associated with the suppression of Th2 cytokines; however, no effects are exerted sometimes^42^. Suppression of IL-4, IL-5, and IL-10 was associated with low IgE titres and reduced eosinophil, lymphocyte, and monocyte counts. Activation of Th1 cells was associated with a higher neutrophil count^43–45^; therefore, the present results suggest that *L*. *chungangensis* CAU 28 cream cheese affects AD recovery through restoration of the Th2/Th1 balance.

The gut microbial community was analysed to investigate the association between immunomodulation and gut microbiota, revealing higher levels of bacteria of the genus *Faecalibacterium* and lower levels of the *Ruminococcus*, *Lactobacillus*, and *Bacteroides* genera in the positive control group compared with the CAU 28 cream cheese group. These strains are considered important gut microbiota in AD. High levels of *Faecalibacterium prausnitzii* are associated with exacerbated inflammation, reductions in SCFA levels, and altered Th2-type immune responses^40^. *Bacteroides fragilis* induces Tregs and IL-10, while *L*. *plantarum* and *L*. *rhamnosus* GG produces small-molecule immunomodulators, such as the anti-inflammatory cytokine, IL-10^28,46^. Genus *Ruminococcus* is associated with high IgE levels^39^. Interestingly, a high bacterial count of *Akkermansia* spp. (family *Verrucomicrobiales*) was observed in the CAU 28 cream cheese group and the negative control group. These strains are mucin-degrading and are reportedly associated with energy metabolism and homeostasis; however, their exact functions in the present context remains unclear^47^. Nevertheless, previous studies have reported that an abundance of *Akkermansia* spp. bacteria is associated with anti-inflammatory responses in diseases including type 1 and 2 diabetes mellitus^48,49^, inflammatory bowel disease^50^, and obesity^51^.

In the present study, *L*. *chungangensis* CAU 28 and CAU 28 cream cheese modulate gut microbiota, followed by metabolite production and modulation of the immune response for therapeutic effects in AD. These results are concurrent with previous results wherein pro- and prebiotic administration (foodstuffs) and faecal microbiota transplantation (FMT) are considered therapeutic, since these modulate the gut microbiota while simultaneously restoring physiological functions via production of metabolites owing to the stimulation as beneficial bacteria growth^52,53^. In the present study, concurrently, treatment with CAU 28 cream cheese yielded superior results compared with CAU 28 alone, probably because of the presence of additional compounds in *L*. *chungangensis* CAU 28 cream cheese. Previously, 14 SCFAs were detected in *L*. *chungangensis* CAU 28 cream cheese via gas chromatography analysis (data not shown). Of the SCFAs detected, oleic acid (omega-9) stabilizes the lipid lamellar sheet, thereby potentially reducing water loss from the skin^54^. In addition, linoleic acid (omega-6) plays a role in skin barrier permeability and can modulate immune responses associated with inflammatory dermatoses, psoriasis, and AD^55^. Furthermore, α-linoleic acid (omega-3) is an important regulator of transcription factors related to lipid metabolism, inflammation, immune regulation, and skin barrier homeostasis^55,56^. Therefore, *L*. *chungangensis* CAU 28 cream cheese is probably more effective in treating AD than purified *L*. *chungangensis* CAU 28 because it provides these SCFAs.

**Figure 10:**
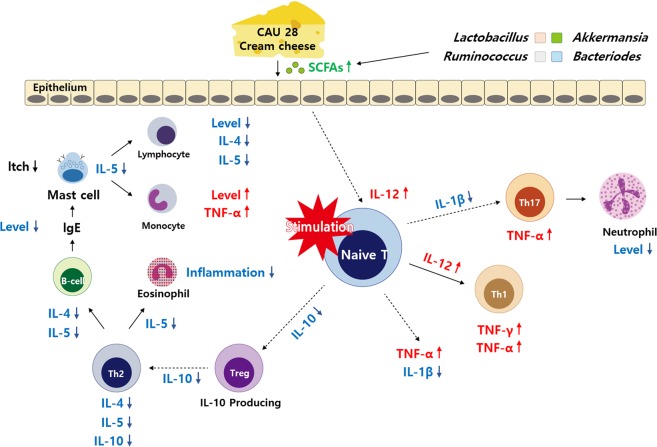
Overview of the mechanism underlying the efficacy of *Lactobacillus chungangensis* CAU 28 cream cheese in treating atopic dermatitis (AD). Summary of the present results and a proposed mechanism indicating the roles of the immune system, gut microbiota, and short-chain fatty acids in AD. The mechanistic links are represented by solid lines (—, direct links) and dotted lines (—, speculative links).

In conclusion, treatment of AD with *L*. *chungangensis* CAU 28 cream cheese yielded beneficial results, including the suppression of Th2 immune responses, a gut microbiota profile similar to that of the negative control group, and high SCFA levels (Fig. 10). Together, these results provide a deeper insight into the immune mechanisms, gut microbiota, and SCFA modulation in AD. Moreover, these data suggest that oral administration of the dairy product, *L*. *chungangensis* CAU 28 cream cheese has potential applications in preventing or treating AD. *L*. *chungangensis* CAU 28 cream cheese thus serves as an alternative treatment for AD, although complementary clinical trials are necessary.

## Methods

### Ethics and animals

Five-week-old female BALB/c mice (n = 50) were purchased from Central Lab Animal Incorporation (Korea) and allowed to acclimate for 1 week before the start of the experiments. Five mice were housed in a cage with ad libitum access to a nutritionally complete chow and water under a 12:12-h light/dark cycle. The temperature and humidity were maintained at 24 °C ± 2 °C and 55% ± 10%, respectively. Animal studies were performed in accordance with the Korean Food and Drug Administration guidelines. Samples were collected from mice under the animal ethical guidelines mandated by the protocol presented to and approved by the Chung-Ang University Institutional Animal Care and Use Committee of the Laboratory Animal Research Center (No. 2017–00044). Mice were randomly assigned to five groups (n = 10/group): (1) negative control (non-OVA sensitization + PBS), (2) positive control (OVA sensitization + PBS), (3) BB (OVA sensitization + BB), (4) *L*. *chungangensis* CAU 28 (OVA sensitization + *L*. *chungangensis* CAU 28), and (5) CAU 28 cream cheese group (OVA sensitization + cream cheese derived *L*. *chungangensis* CAU 28). To induce AD in mice via skin sensitization, the dorsal skin of each mouse was shaved using electric clippers and hair removal cream. OVA grade V (Sigma-Aldrich, USA) (50 mg/mL) with alum (Sigma-Aldrich) in PBS was intraperitoneally injected on days 7, 21, 35, and 49, and mice were epicutaneously sensitized for 8 weeks, as previously described^57,58^ (Fig. S3). Freeze-dried *L*. *chungangensis* CAU 28 (1 × 10^10^ colony forming units/mouse) and cream cheese prepared with *L*. *chungangensis* CAU 28 (1.4 g/kg/mouse), were dissolved in 200 μl of sterilized water, and administered via an oral gavage. The negative control group was treated with an equal volume of PBS for 8 weeks. BB, the positive treatment control, was administered orally once a day for 8 weeks (0.5 mg/kg). Thereafter, mice were euthanised, and the dorsal skin, ileum, spleen, and blood were harvested for further analyses.

### DNA sample preparation, high-throughput sequencing, and microbiome analysis

Faecal samples, collected after 16 weeks of treatment, from individual mice in sterile 2 mL microcentrifuge tubes, were immediately transported on ice, and stored at −80 °C. DNA was extracted using a FastDNA SPIN kit for bacterial DNA (MP Biomedicals, USA) in accordance with the manufacturer’s instructions. PCR amplification of the 16 S rRNA gene V3–V4 region was performed using MiSeq-based high throughput sequencing (Illumina, USA). Samples that passed quality control were used to construct a library. After sequencing, adapter sequences were removed from the resulting data using the programs Scythe (v 0.994; https://github.com/vsbuffalo/scythe) and Sickle (https://github.com/vsbuffalo/scythe). Sequences were trimmed to eliminate short reads (<36 bp), extra-long tails, chimeric reads, and noise sequences, using CD-HIT-OTU (http://weizhong-lab.ucsd.edu/cd-hit-otu)^59^. The minimum quality score and length of merged reads were >20 and 300 bp, respectively. Remaining representative sequences were clustered and operational taxonomic units (OTUs) defined at a cutoff of 97% similarity, using CD-HIT-OTU. Microbiome analysis was conducted upon defining OTUs, using UCLUST and taxonomic assignment was achieved using QIIME (Quantitative Insight Into Microbial Ecology; v.1.9.1)^60^ by screening data in the 16 S rRNA sequence database of the Ribosomal Database Project (RDP; release 11.0, update 5) (http://rdp.cme.msu.edu/). *α*-diversity was calculated from the following indices: observed number of species, Chao1, and ACE (richness estimators); and Shannon, Simpson, and InvSimpson (estimators of sample diversity). The ordinate function supported constrained correspondence analysis (CCA), detrended correspondence analysis (DCA), and redundancy analysis (RDA). For CCA, DCA, and RDA, ordination was based on an evaluation of abundance values, but not ecological distance. The CCA and DCA were used to determine the correlation among groups, while RDA was used to determine that explained most of the variance in the groups. Moreover, correlation of bacterial taxa at the family level with data from flow cytometry analysis (CD 86 and CD 274) were determined using frequency tables for bacteria at various levels of taxonomic resolution analysed using a Spearman non-parametric rank correlation matrix.

### Analysis of short-chain fatty acids in faecal samples

SCFA and lactic acid content in mouse faecal samples were analysed via HPLC (Ultimate 3000, Thermo Dionex, USA). Briefly, faecal samples (100 mg) were homogenized for 10 min in 0.005 M aqueous NaOH containing 5 μg/ml caproic acid (Sigma-Aldrich), and centrifuged at 12,000 × *g* for 20 min at 4 °C. Aliquots of supernatants were transferred to disposable glass centrifuge tubes (Corning, USA) and stored at −80 °C until required for HPLC analysis. SCFAs were separated using an Aminex 87 H column (300 × 10 mm; Bio-Rad, USA), using an isocratic 0.01 N H_2_SO_4_ mobile phase (Fluka, USA) at a flow rate of 0.5 ml/min and a temperature of 40 °C. Volatile organic acid (VOA) mixtures (including formic, acetic, propionic, isobutyric, and butyric acid) and lactic acid were detected at 210 nm, using an RI-detector (ERC, RefractoMax 520, Japan). Acids were identified via comparison with the corresponding standards: (VOA mixture, 10 mM; AccuStandard FAMQ-004, USA).

### Blood and serum cytokine analysis

Blood was collected via puncturing of the retro-orbital sinus via the medial canthus of the eye. Whole blood was collected in spray-dried EDTA tubes (Green Cross Laboratories, Korea) to enumerate eosinophils and immediately mixed to prevent coagulation. Blood was then coagulated for 1 h at 4 °C and centrifuged for 1 h at 5,000 × *g* for IgE and cytokine (IL-4, IL-5, IL-10, IL-12, IL-1β, TNF-α, and IFN-γ) analyses. Serum was stored at −80 °C until analysis. Cytokine levels were measured using ELISA kits (R&D systems, USA) in accordance with the manufacturer’s instructions. Absorbance was measured at 450 nm, using a microplate reader.

### Analysis of mRNA expression in ileal tissue recovered from formalin-fixed paraffin-embedded (FFPE) slides

Twenty-micrometre-thick unstained ileal tissue sections were obtained using a microtome (Leica, Germany) for RNA extraction and deparaffinized using Deparaffinization Solution (Qiagen, USA) at 56 °C for 3 min, followed by cooling at room temperature, and completely digested by proteinase K at 56 °C for 15 min and at 80 °C for 15 min. Thereafter, DNase I was used to eliminate DNA. RNA from FFPE samples were extracted using RNeasy FFPE kit (Qiagen) in accordance with the manufacturer’s protocol (RNeasy FFPE handbook). The quantity of RNA was measured using NanoQuant spectrophotometer (Infinite 200; Tecan, Switzerland). Thereafter, first-strand cDNA synthesis was performed using 1 μg of total RNA with oligo dT primer (50 μM), using the PrimeScript 1st strand cDNA synthesis kit (Takara, Japan). Target mRNA (IL-4, IL-5, IL-10, IL-12, IL-1β, TNF-α, and IFN-γ) expression levels were analysed using ABI Fast 7500 real-time system instruments (Applied Biosystems, USA) and were normalised relative to the expression levels of housekeeping gene (36B4; ribosomal protein RPLP0), using. the comparative Ct method and the 2^−ΔΔCt^ formula.

### Flow cytometry analysis

Harvested spleens were homogenised for 3 min at room temperature in 50-mm tissue culture Petri dishes (Nunclon, Denmark) in ammonium chloride potassium (ACK) lysing buffer (Gibco, USA) to lyse erythrocytes. Samples were filtered using a cell strainer (SPL, Korea) and washed in 2% RPMI (Hyclone, USA). After centrifugation at 2,000 × *g* for 10 min at 4 °C, supernatants were discarded, and pellets resuspended in 2% RPMI. Splenocytes were enumerated, and their viability was assessed using Trypan Blue. Cells were diluted to 2.0 × 10^6^ cells per tube and incubated on ice with phycoerythrin (PE)-labelled anti-mouse antibodies: CD80, CD86, CD273, or CD274 (BD Pharmingen, USA) for 20 min. Cell surface expression of different molecules was assessed by recording at least 10,000 events, using a flow cytometer (FACSCalibur; Becton Dickinson, USA). Mean fluorescence intensity (MFI) data were analysed using a FACSCalibur instrument and Cell Quest software (version 6.0).

### Evaluation of dermatitis scores

Dermatitis severity in the dorsal skin of OVA-sensitized AD mice was evaluated based on the occurrence of erythema, dryness, and scratching behaviour within 15 min after 1-, 8-, and 16-week sensitization. Severity scores were 0 (none), 1 (mild), 2 (moderate), or 3 (severe). Final scores were calculated as the sum of individual scores.

### Histological analysis

FFBE dorsal skin and ileal tissue of the euthanised mice were used for histological analysis. Tissues were fixed in 10% formalin in PBS and embedded in paraffin. Tissue sections were cut at 4–5-μm thickness and stained with haematoxylin and eosin for histological general analysis, and with Toluidine Blue and Congo Red for mast cell and eosinophil evaluation, respectively. All fields of Toluidine Blue-stained ileum sections were observed to enumerate mast cells at 400× magnification. Five random fields of Toluidine Blue-stained dorsal skin sections were chosen for mast cell quantification and twenty random fields of Congo Red-stained dorsal skin sections were chosen for eosinophil quantification at 400× magnification.

### Statistical analysis

Statistical analysis was performed using GraphPad Prism (v.7.0) and data are presented as mean ± standard error of the mean (SEM) values. To evaluate relative differences, statistically significant differences between groups were determined using the non-parametric Kruskal-Wallis test for microbial phyla and genera and one-way analysis of variance for multiple-group comparisons in cytokine, flow cytometry, blood, histologic, and SCFA analyses. *P*-values less than 0.05 were considered significant.

### Ethics approval

Samples were collected from mice in accordance with the animal ethical guidelines mandated by the protocol presented to and approved by the Chung-Ang University Institutional Animal Care and Use Committee of the Laboratory Animal Research Center (No. 2017–00044).

## Supplementary information

Dataset 1

## Data Availability

The nucleotide sequences are deposited in GenBank under accession number SRP149062.
