# Author Correction: Cream Cheese-Derived *Lactococcus chungangensis* CAU 28 Modulates the Gut Microbiota and Alleviates Atopic Dermatitis in BALB/c Mice

**DOI:** 10.1038/s41598-019-42748-z

**Published:** 2019-05-01

**Authors:** Jong-Hwa Kim, Kiyoung Kim, Wonyong Kim

**Affiliations:** Department of Microbiology, Chung-Ang University College of Medicine, Seoul, Korea

Correction to: *Scientific Reports* 10.1038/s41598-018-36864-5, published online 24 January 2019

This Article contains a typographical error in the legends of Figures 3, 4, 5, 6, 8, 9 and 10, where “*Lactobacillus chungangensis*” should read “*Lactococcus chungangensis*”.

