# Supplementary material for: Cream Cheese-Derived Lactococcus chungangensis CAU 28 Modulates the Gut Microbiota and Alleviates Atopic Dermatitis in BALB/c Mice
Source: Sci Rep. 2019 Jan 24;9:446. doi: 10.1038/s41598-018-36864-5 (PMC6345912; doi:10.1038/s41598-018-36864-5)
Supplement: Supplementary file 1 — Dataset 1 [file 41598_2018_36864_MOESM1_ESM.doc]

**Supplementary information for manuscript:**

**Cream Cheese-Derived *Lactococcus chungangensis* CAU 28 Modulates the Gut Microbiota and Alleviates Atopic Dermatitis in Balb/c Mice**

Jong-Hwa Kim, Kiyoung Kim, and Wonyong Kim^*^

**Table S1. Simpson and Shannon-Wiener diversity indices.** Alpha diversity expressed as OTUs (defined as 97% sequence similarity), and Simpson and Shannon-Wiener Indexes in bacterial communities from each group. (-), Negative control group; (+), Positive control group; CC, cream cheese; BB, bepotastine besilate

| **No.** | **OTUs** | | | | | **Simpson Index** | | | | | **Shannon–Wiener index** | | | | |
| --- | --- | --- | --- | --- | --- | --- | --- | --- | --- | --- | --- | --- | --- | --- | --- |
|  | **(-)** | **(+)** | **CAU 28** | **CAU 28 CC** | **BB** | **(-)** | **(+)** | **CAU 28** | **CAU 28 CC** | **BB** | **(-)** | **(+)** | **CAU 28** | **CAU 28 CC** | **BB** |
| 1 | 266 | 311 | 277 | 324 | 335 | 0.923 | 0.84 | 0.834 | 0.926 | 0.882 | 4.847 | 4.087 | 3.702 | 5.198 | 4.554 |
| 2 | 236 | 339 | 221 | 273 | 325 | 0.864 | 0.882 | 0.863 | 0.866 | 0.903 | 4.287 | 5.985 | 4.012 | 4.291 | 4.896 |
| 3 | 341 | 364 | 274 | 302 | 407 | 0.957 | 0.96 | 0.934 | 0.931 | 0.974 | 5.742 | 4.71 | 5.413 | 5.377 | 6.273 |
| 4 | 332 | 347 | 240 | 305 | 339 | 0.956 | 0.869 | 0.829 | 0.907 | 0.914 | 5.737 | 5.905 | 3.615 | 4.765 | 5.028 |
| 5 | 328 | 316 | 227 | 337 | 367 | 0.865 | 0.852 | 0.767 | 0.92 | 0.886 | 4.275 | 4.649 | 3.155 | 5.092 | 4.808 |
| 6 | 291 | 409 | 254 | 290 | 298 | 0.87 | 0.89 | 0.888 | 0.93 | 0.901 | 4.172 | 4.516 | 4.28 | 4.968 | 4.783 |
| 7 | 305 | 314 | 273 | 340 | 317 | 0.976 | 0.802 | 0.824 | 0.906 | 0.939 | 6.327 | 5.206 | 3.542 | 4.807 | 5.295 |
| 8 | 260 | 339 | 180 | 385 | 312 | 0.928 | 0.876 | 0.868 | 0.955 | 0.963 | 5.236 | 4.023 | 4.086 | 5.919 | 5.999 |
| 9 | 240 | 281 | 175 | 353 | 315 | 0.892 | 0.769 | 0.727 | 0.975 | 0.939 | 4.563 | 4.562 | 3.521 | 6.378 | 5.586 |
| 10 | 229 | 398 | 303 | 326 | 303 | 0.882 | 0.958 | 0.822 | 0.941 | 0.941 | 4.787 | 3.618 | 3.999 | 5.718 | 5.291 |
| **Mean**  **± SD** | 282.80  ± 42.33 | 341.80  ± 39.85 | 242.40  ± 42.26 | 323.50  ± 32.64 | 331.80  ± 33.11 | 0.91  ± 0.04 | 0.87  ± 0.06 | 0.84  ± 0.06 | 0.93  ± 0.03 | 0.92  ± 0.03 | 4.50  ± 0.74 | 4.73  ± 0.78 | 3.93  ± 0.62 | 5.25  ± 0.62 | 5.25  ±0.56 |

**Table S2. Taxonomy of abundant gut microbiota identified in the experimental groups. Relative abundance of statistically significant (*p* < 0.001) bacteria in microbiota. (-), Negative control group; (+), Positive control group; CC, cream cheese; BB, bepotastine besilate**

| **Phylum** | **Order** | **Genus** | **Average (%)** | | | | | ***p* value** |
| --- | --- | --- | --- | --- | --- | --- | --- | --- |
|  |  |  | **(-)** | **(+)** | **CAU 28** | **CAU 28 CC** | **BB** |  |
| Bacteroidetes | Bacteroidales | *Alistipes* | 1.056 ± 0.32 | 0.393 ± 0.13 | 0.075 ± 0.09 | 1.058 ± 0.47 | 1.823 ± 0.61 | < 0.0001 |
|  |  | *Bacteroides* | 0.870 ± 0.57 | 0.673 ± 0.27 | 0.140 ± 0.10 | 1.402 ± 0.65 | 0.770 ± 0.24 | < 0.001 |
|  |  | *Prevotella* | 1.049 ± 0.35 | 3.595 ± 0.52 | 0.017 ± 0.01 | 1.154 ± 0.59 | 2.222 ± 0.84 | < 0.0001 |
| Firmicutes | Lactobacillales | *Lactobacillus* | 21.359 ± 2.29 | 11.736 ± 3.09 | 22.450 ± 4.81 | 23.623 ± 4.46 | 18.501 ± 4.02 | < 0.0001 |
|  | Clostridiales | *Faecalibacterium* | 0.062 ± 0.06 | 0.635 ± 0.23 | 0.000 ± 0.00 | 0.196 ± 0.10 | 0.552 ± 0.29 | < 0.0001 |
|  |  | *Ruminococcus* | 0.603 ± 0.31 | 0.068 ± 0.04 | 0.480 ± 0.19 | 0.414 ± 0.26 | 0.155 ± 0.06 | < 0.001 |
| Verrucomicrobia | Verrucomicrobiales | *Akkermansia* | 16.913 ± 7.27 | 0.697 ± 1.64 | 0.308 ± 0.45 | 8.175 ± 9.57 | 0.161 ± 0.18 | < 0.0001 |

**FIG. S1. Relative abundance of bacterial taxonomy levels in the gut microbiota.** Differences in the phylum level proportions and relative abundances at phylum, family, and genus levels in each mouse in each experimental group are shown.


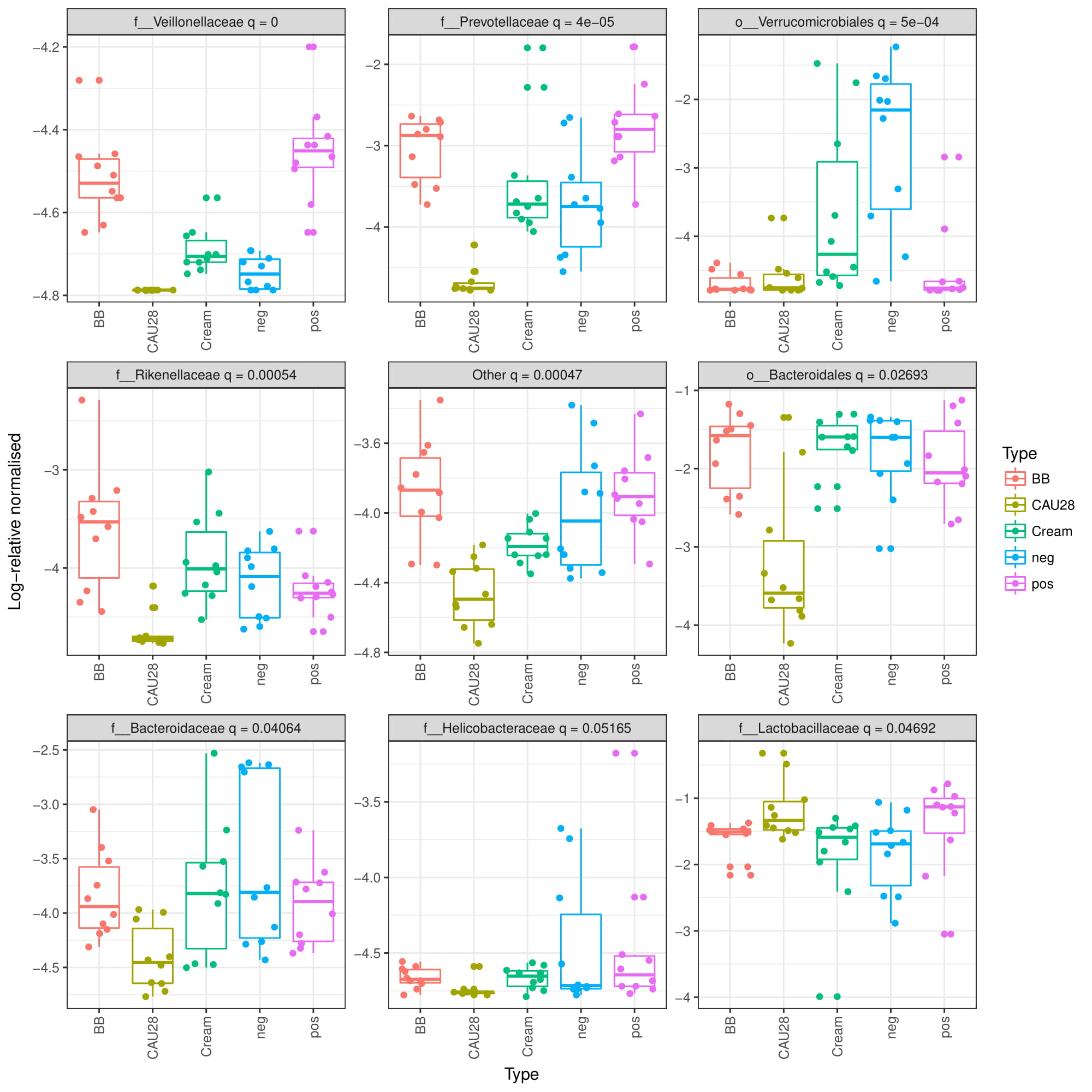


**FIG. S2. Difference in abundances among groups.** Normalization of zero or small numbers of proportions of relative abundances at the family level. Box plots showing the frequencies of statistically significant bacterial families. neg, negative control group; pos, positive control group; cream, CAU 28 cream cheese; BB, bepotastine besilate.

**FIG. S3. Experimental process.** Experimental time line from the induction of atopic dermatitis in mice by ovalbumin (OVA) sensitization to analysis.
